# Apomorphine Suppresses the Progression of Steatohepatitis by Inhibiting Ferroptosis

**DOI:** 10.3390/antiox13070805

**Published:** 2024-07-02

**Authors:** Hiroshi Maeda, Kouichi Miura, Kenichi Aizawa, Oyunjargal Bat-Erdene, Miho Sashikawa-Kimura, Eri Noguchi, Masako Watanabe, Naoya Yamada, Hitoshi Osaka, Naoki Morimoto, Hironori Yamamoto

**Affiliations:** 1 Department of Medicine, Division of Gastroenterology, Jichi Medical University, 3311-1 Yakushiji, Shimotsuke 329-0498, Tochigi, Japane-noguchi@jichi.ac.jp (E.N.);; 2 Division of Clinical Pharmacology, Department of Pharmacology, Jichi Medical University, 3311-1 Yakushiji, Shimotsuke 329-0498, Tochigi, Japan; 3 Department of Dermatology, Jichi Medical University, 3311-1 Yakushiji, Shimotsuke 329-0498, Tochigi, Japan; 4 Division of Inflammation Research Center for Molecular Medicine, Jichi Medical University, 3311-1 Yakushiji, Shimotsuke 329-0498, Tochigi, Japan; 5 Division of Pediatrics, Jichi Medical University, 3311-1 Yakushiji, Shimotsuke 329-0498, Tochigi, Japan

**Keywords:** steatohepatitis, ferroptosis, apomorphine

## Abstract

The role of ferroptosis in steatohepatitis development is largely unknown. We investigated (1) whether hepatocyte ferroptosis occurs in a gene-modified steatohepatitis model without modifying dietary components, (2) whether ferroptosis occurs at an early stage of steatohepatitis, and (3) whether apomorphine, recently reported as a ferroptosis inhibitor, can ameliorate steatohepatitis. Hepatocyte-specific PTEN KO mice were used. Huh 7 and primary cultured hepatocytes isolated from the mice were used in this study. The number of dead cells increased in 10-week-old PTEN KO mice. This cell death was suppressed by the administration of ferroptosis inhibitor ferrostatin-1 for 2 weeks. Apomorphine also ameliorated the severity of steatohepatitis. Treatment with ferroptosis inhibitors, including apomorphine, decreases the level of lipid peroxidase. Apomorphine suppressed cell death induced by RSL-3 (a ferroptosis inducer), which was not suppressed by apoptosis or necroptosis inhibitors. Apomorphine showed a radical trapping capacity with much more potent activity than ferrostatin-1 and Trolox, a soluble form of vitamin E. In addition, apomorphine activated nrf2 and its downstream genes, including HO-1 and xCT. In conclusion, ferroptosis occurs in steatohepatitis from an early stage in PTEN KO mice. In addition, apomorphine ameliorates the severity of steatohepatitis by inhibiting ferroptosis.

## 1. Introduction

Non-alcoholic fatty liver disease (NAFLD), currently known as metabolic dysfunction-associated steatotic liver disease (MASLD) [1], is the most common liver disease, affecting approximately 38% of the general population worldwide [2]. In addition, 5.27% of the general population is affected by non-alcoholic steatohepatitis (NASH), an advanced form of NAFLD [2]. MASLD is a risk factor not only for liver cirrhosis and hepatocellular carcinoma but also for cardiovascular diseases and extrahepatic malignancies. Consequently, MASLD is recognized as a life-threatening disease. However, treatment agents for MASLD are not available.

Multiple types of hepatocyte death have been noted in steatohepatitis, including apoptosis, necrosis, necroptosis, and ferroptosis [3]. Among these types of cell death, apoptosis has been widely studied in both human and animal models. In humans, the number of apoptotic cells increases with the progression of steatohepatitis [4]. The severity of steatohepatitis was reduced with a lower number of apoptotic cells in mice in which key molecules for apoptosis, including caspase 3 and caspase 8, were deleted [5,6]. However, pan-caspase inhibitors failed to show beneficial effects in patients with NAFLD [7]. Necrosis/necroptosis is another form of cell death related to the progression of NAFLD. The number of necroptotic hepatocytes increases as the fibrosis stage advances in human NASH [8]. In addition, the expression of RIPK3, a key molecule in necroptosis, is increased in the liver of animal models and in patients with NAFLD [9]. Interestingly, necroptosis markers are barely detected in patients with fibrosis stage 0, an early stage of NAFLD [8]. These data suggest that apoptosis and necrosis/necroptosis seem to be modes of cell death that occur at an advanced stage of steatohepatitis. Therefore, we must pay attention to cell death that occurs from the early stage of steatohepatitis. 

Ferroptosis is a newly recognized form of cell death characterized by the iron-dependent accumulation of lipid hydroperoxides at lethal levels [10]. Recent studies have shown that ferroptosis is associated with various liver diseases [11]. Excessive iron deposition and oxidative stress are well-known features of NAFLD [12]. Indeed, antioxidant treatment and phlebotomy have been shown to have beneficial effects on human NAFLD in some studies [13,14]. In animal models, ferroptosis inhibitors have been shown to protect against the development of diet-induced steatohepatitis [15,16,17]. In addition, ferroptosis occurs during the early phase of steatohepatitis [17]. However, the aforementioned studies used methionine-and/or choline-deficient diets [15,16,17], which cause body weight loss, opposite to the features of typical MASLD. Thus, it is necessary to investigate whether ferroptosis occurs universally in steatohepatitis without modifying dietary components.

Apomorphine is an agent used in the treatment of Parkinson’s disease [18,19], in which dopamine function is decreased in the brain. Apomorphine has multiple functions as a dopamine receptor agonist, radical trapping agent [20], and activator of nuclear factor-erythroid 2-related factor 2 (nrf2) [21]. We found that fibroblasts isolated from patients with mitochondrial diseases were vulnerable to oxidative stress and that apomorphine rescued cells from ROS-induced cell death [22]. In addition, we demonstrated that ferroptosis causes cell death [23]. However, there are limited data on the effects of apomorphine on hepatocytes and liver diseases, including steatohepatitis. 

In the present study, we aimed to investigate the following: (1) whether ferroptosis occurs in hepatocyte-specific PTEN KO mice, in which steatohepatitis is noted without modifying dietary components; (2) whether ferroptosis occurs from an early stage of steatohepatitis; and (3) whether apomorphine can act as a ferroptosis inhibitor in hepatocytes and in a steatohepatitis model.

## 2. Materials and Methods

### 2.1. Reagents 

The following reagents were used in the present study: (1S,3R)-RSL-3 (RSL-3, #19288, Cayman, Ann Arbor, MI, USA), ferrostatin-1 (#HY-100579, Med Chem Express, South Brunswick, NJ, USA), GSK872 (a receptor-interacting protein kinase 3 [RIP3K] inhibitor, #S8465, Selleckchem, Houston, TX, USA), Z-VAD-FMK (a pan-caspase inhibitor, #S7023, Selleckchem, Houston, TX, USA), rotigotine (#R9281, Sigma-Aldrich Japan, Tokyo, Japan), MTT (#11465007001, Sigma-Aldrich Japan), BOBIPY581/591 (#D3861, Thermo Fisher, Waltham, MA, USA), Hoechst33342 (#62242, Thermo Fisher), propidium iodide (PI, #P4170, Sigma-Aldrich), apomorphine (#013-18323, FUJIFILM, Osaka, Japan), and dimethyl sulfoxide (DMSO, #037-24053, FUJIFILM).

### 2.2. Animals 

We used hepatocyte-specific PTEN knockout (PTEN KO) mice in the present study because dietary components, such as iron, may affect ferroptosis. The PTEN KO mice were generated as previously reported [24]. Albumin-Cre recombinase-negative PTEN flox/flox mice (littermates) were used as controls. All mice were on a C57Bl6 background and 8-week-old male mice were used. The mice were given standard chow (MFG-LID, Oriental Yeast Co., Ltd., Tokyo, Japan) and had ad libitum access to food and water until the end of the experiments under specific-pathogen-free conditions. The number of mice used in the present study is indicated in the figure legends (n = 3–13). The treatment agents were intraperitoneally injected daily for 14 days. Reagents were dissolved in 3% DMSO and the doses of each reagent were as follows according to the published papers [16,25]: ferrostatin-1 (5 mg/kg), apomorphine (0.5 mg/kg), GSK872 (2 mg/kg), and rotigotine (0.5 mg/kg). Bodyweight and diet were also monitored. After 2 weeks of treatment, the mice were killed, and harvested samples were stored at −80 °C until use. The animal experiments were approved by the Review Board of Jichi Medical University (20061; Tochigi, Japan). All animals received humane care according to the criteria outlined in the *Guide for the Care and Use of Laboratory Animals* [26] published by the National Academy of Science, as well as the policies of our institution. 

### 2.3. Cell Culture and Cell Death Assay

Huh 7, a hepatocellular carcinoma cell line, and primary cultured hepatocytes isolated from the control and PTEN KO mice were used. Hepatocytes were isolated as previously reported [27]. Huh 7 cells and primary hepatocytes were cultured in Dulbecco’s modified Eagle medium (DMEM) (FUJIFILM, Japan) supplemented with 10% fetal bovine serum and antibiotics. Huh 7 cells seeded onto appropriate dishes/plates were used when the cell density reached 60% confluency. For primary cultured hepatocytes, 1 × 10^5^ and 2 × 10^4^ cells were seeded onto 24-well dishes and 96-well plates, respectively. In the cell death assay, serum was starved and treatment agents (inhibitors of cell death) were added 1 h before treatment with RSL-3 (a ferroptosis inducer). The cells were cultured with reagents at the concentrations indicated in the figure legends for 24 h. The supernatants were subjected to lactate dehydrogenase (LDH) measurement using FUJI DRI-CHEM SLIDE (FUJIFILM, Japan) according to the manufacturer’s instructions. The cell numbers were counted using an MTT assay. Cell morphology was observed under a phase-contrast microscope (Olympus, Tokyo, Japan). Cell death was assessed using PI. Hoechst 33342 was used for nuclear staining.

For the peroxidase analysis, Huh 7 cells were labeled with 5 μM BOBIPY581/591 dissolved in DMEM supplemented with 0.1% bovine serum albumin for 1 h before RSL-3 treatment. The nuclei were stained with Hoechst 33342. Fluorescence imaging was performed using an FV10i confocal laser-scanning microscope (Olympus, Tokyo, Japan).

### 2.4. Histology

Hematoxylin and eosin (H&E) and Oil Red O staining were performed according to published protocols [27]. The NAFLD activity score was evaluated according to a published protocol [28]. Ten randomly selected fields were subjected to NAFLD activity scoring (mice n = 5 from each group). Immunostaining for TUNEL (R&D Systems, Minneapolis, MN, USA), RIP3 (#ab62344, Abcam Japan, Tokyo, Japan), and albumin (#16475-1-AP, Proteintech Japan, Tokyo, Japan) was performed as previously described [27]. To assess cell death in the liver, PI was injected into the tail vein according to a published protocol [17]. Liver samples were harvested 10 min after injection and snap-frozen in liquid nitrogen. Ten randomly selected fields were subjected to PI-positive cell counting (mice n = 3–4 per group).

### 2.5. Quantitative Real-Time Polymerase Chain Reaction (PCR) 

RNA was extracted from the liver and cells using TRI Reagent^®^ (Sigma-Aldrich Japan, Japan). The extracted RNA was reverse transcribed into cDNA. The cDNA was then subjected to PCR using the primers listed in Supplemental Table S1 and the TB Green^®^ Primer Ex Taq (Takara Bio Inc., Shiga, Japan). Gene expression was normalized to that of 18S RNA as an internal control.

### 2.6. Measurements 

Serum aspartate aminotransferase and alanine aminotransferase levels were assessed using FUJI DRI-CHEM SLIDE according to the manufacturer’s instructions. A total of 10 mg and 30 mg of liver tissue was used to measure iron (#MAK025, Sigma-Aldrich) and malondialdehyde (MDA, #M496, Dojindo Molecular Technologies Inc., Kumamoto, Japan), respectively. The liver samples were processed according to the manufacturer’s instructions. Briefly, the liver tissue was homogenized in the supplied buffer, and the supernatant was subjected to total iron and Fe^2+^ analyses after centrifugation. For the measurement of MDA, the liver tissues were homogenized in an antioxidant buffer, and the supernatants were subjected to measurement after centrifugation. Absorbance was evaluated using a Multiskan FC (Thermo Fisher) with the indicated filters, and the concentration was calculated using the pseudo-endpoint method. For the radical trapping assay, the 2,2-diphenyl-1-1picrylhydrazyl (DPPH) antioxidant assay kit (#343-09563, Dojindo) was used according to the manufacturer’s instructions.

### 2.7. Western Blotting

Nuclear proteins were isolated from Huh 7 cells according to the manufacturer’s instructions (Abcam, ab113474). After treatment with the sample buffer, 10 mg of protein was subjected to western blotting. The proteins were separated using a proper concentration of sodium dodecyl sulfate–polyacrylamide gel and transferred to nitrocellulose membranes, followed by incubation with an nrf2 antibody (#ab137550, Abcam Japan). Histone (#ab21054, Abcam, Japan) was used as an internal control for the nuclear fractions.

### 2.8. Immunocytochemistry

The cells were rinsed with PBS, fixed with 4% paraformaldehyde, and permeabilized with 0.1% Triton X for 5 min. After treatment with a commercially available blocking agent (#06349-64, NAKARAI TESQUE, Kyoto, Japan) for 1 h, the cells were incubated with an anti-nrf2 antibody (#ab137550, Abcam Japan) overnight at 4 °C. After removing the antibody, the cells were incubated with a fluorescent and Hoechst33342 for 1 h. Cells were captured using a fluorescent microscope. 

### 2.9. Statistical Analysis

STATA (version 17.0; STATA Corporation, College Station, TX, USA) was used for the statistical analyses. All data are presented as the mean ± standard deviation. Statistical significance was determined using the Mann–Whitney U test or Student’s *t*-test. A one-way ANOVA was used to assess body weight and diet intake. *p*-values of <0.05 were considered to indicate statistical significance.

## 3. Results

### 3.1. Ferroptosis Occurred in PTEN KO Mice on a Standard Diet

**Figure 1:**
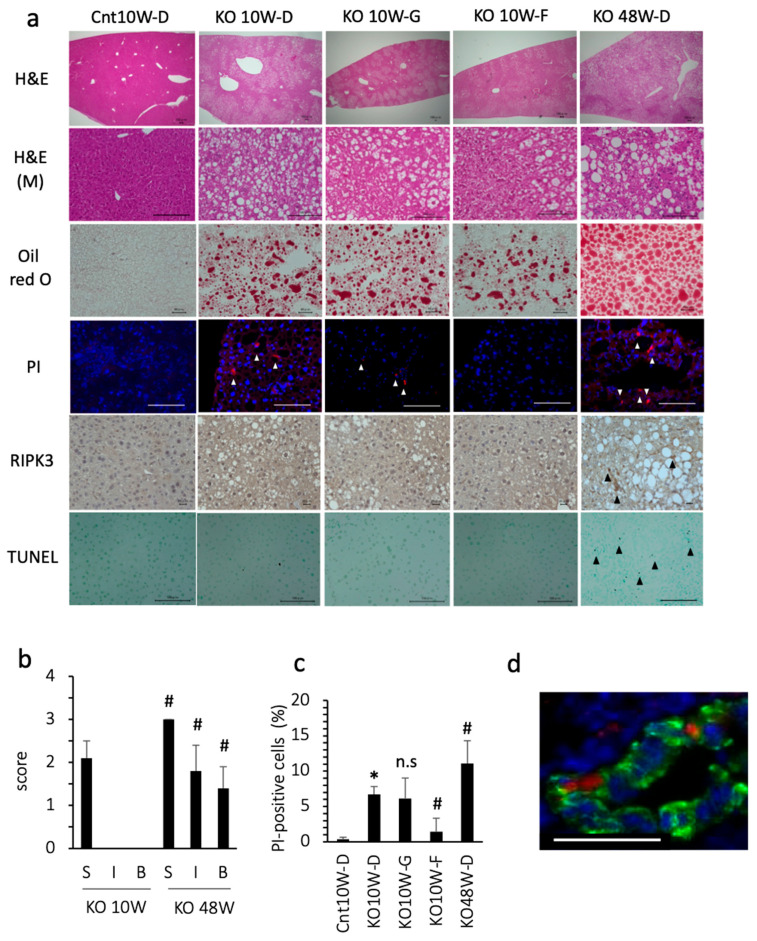
Histological assessment of the liver. Representative photos are shown from each group. Mice were treated with each reagent for 2 weeks and sacrificed at 10 weeks (10W) or 48 weeks (48W) of age. Cnt, control mice: KO, PTEN KO mice. D, DMSO: G, GSK872; F, ferrostatin-1. (**a**) Hematoxylin staining (H&E, bar 100 μm); H&E (M) indicates magnified photographs (bar 100 μm). Oil red O staining (bar 40 μm), PI staining (white arrowheads, bar 100 μm), and immunohistochemical staining of RIPK3 (black arrowheads, bar 20 μm) and TUNEL (black arrowheads, bar 100 μm) were performed. (**b**) NAFLD activity score. Few inflammatory foci and little hepatocyte ballooning were observed in the PTEN KO mice at 10 weeks of age. # KO10W-D vs. KO48W-D (each factor was statistically significant, *p* < 0.05). S, steatosis; I, inflammatory cell foci, B, ballooning of hepatocytes. (**c**) The number of PI-positive cells. * Cnt10W-D vs. KO10W-D (statistically significant, *p* < 0.05); # vs. KO10W-D (statistically significant, *p* < 0.05); n.s, not significant. (**d**) Immunofluorescent staining for albumin (Green), PI (Red), and Hoechst33342 (Blue). Bar 50 μm.

At 10 weeks of age, the PTEN KO mice fed standard chow showed moderate grade steatosis, but few inflammatory cell foci and little hepatocyte ballooning (Figure 1a,b). Even under less inflammatory conditions, the number of dead cells, assessed using PI staining, was significantly increased in comparison with the control mice (Figure 1a,c). Approximately 20% of the PI-positive cells expressed albumin, indicating that dead cells included hepatocytes (Figure 1d). Because PI is used as a necrosis/necroptosis marker, we tested whether GSK 872 (a necroptosis inhibitor) could reduce the number of dead cells. However, GSK872 treatment did not reduce the number of dead cells. Indeed, there were few necroptotic hepatocytes, assessed using RIPK3 staining (0–1 cell in 1000 hepatocytes), in PTEN KO mice at 10 weeks of age. In addition, there were few apoptotic hepatocytes, assessed using TUNEL staining (1–2 cells in 1000 hepatocytes) in the PTEN KO mice at 10 weeks of age regardless of treatment (Figure 1a). In contrast, the ferroptosis inhibitor, ferrostatin-1, reduced the number of PI-positive cells (Figure 1a,c). These data indicate that ferroptosis occurs in PTEN KO mice without modification of dietary components.

### 3.2. Ferroptosis Occurred in the Early Stage of Steatohepatitis

As previously reported [24], the histological findings of 48-week-old KO mice include severe steatosis, inflammation, and hepatocyte ballooning (Figure 1a,b). Additionally, 48-week-old KO mice showed liver fibrosis and some liver tumors [24]. There was a significant increase in cell death, including apoptosis and necroptosis/necroptosis (Figure 1a,c). The percentage of cells that were positive for PI, TUNEL, and RIPK3 was 11%, 5.0%, and 4.5%, respectively. We also tested ferrostatin-1 for 2 weeks, but ferrostatin-1 did not always reduce the number of PI-positive cells or serum transaminase levels. In contrast with the 48-week-old KO mice, the 10-week-old PTEN KO mice were characterized by no fibrosis or no liver tumors, indicating that the 10-week-old PTEN KO mice were in an early stage of steatohepatitis. These data suggest that ferroptosis, but not necrosis/necroptosis or apoptosis, is the major mode of cell death at an early stage of steatohepatitis in PTEN KO mice. 

### 3.3. Apomorphine Ameliorated Liver Injury

**Figure 2:**
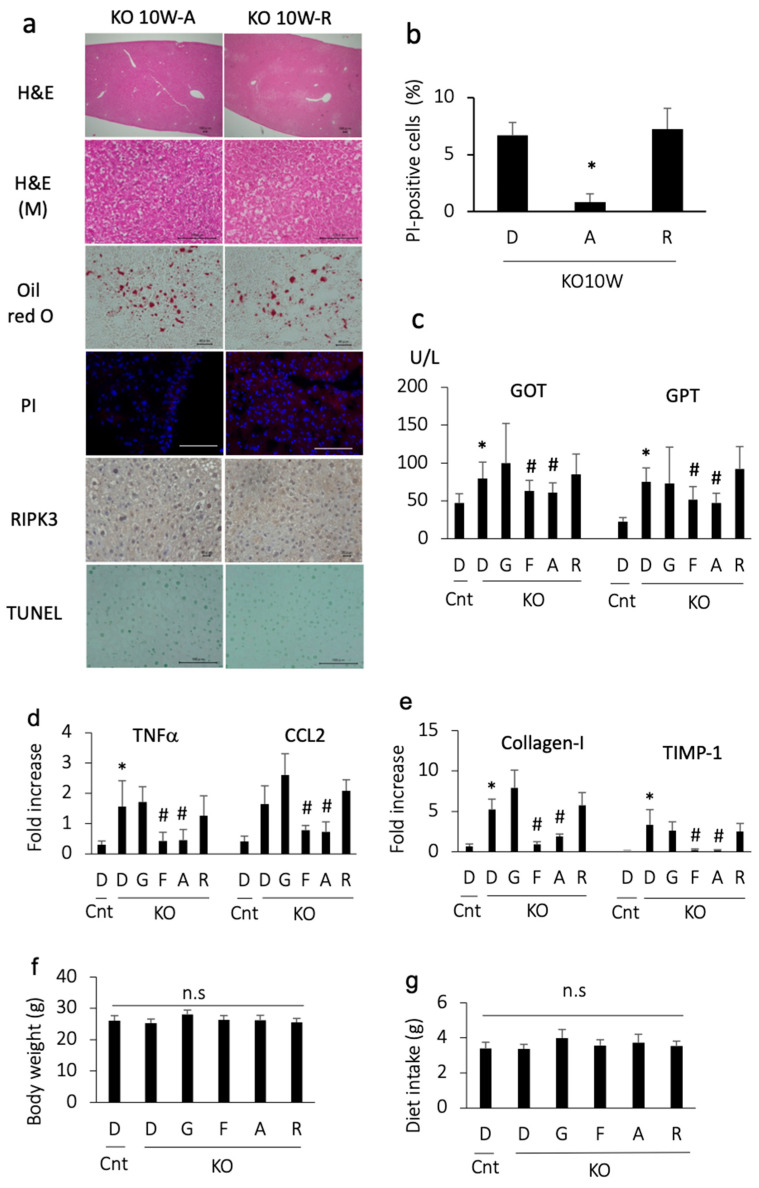
Histological and biochemical assessment in mice treated with each reagent for 2 weeks and sacrificed at 10 weeks of age. Representative photographs are shown from each group. D, DMSO; A, apomorphine; R, rotigotine; G,GSK872; F, ferrostatin-1. Cnt, control mice; KO, PTEN KO mice. * and # indicate statistical significance (*p* < 0.05) relative to the control mice treated with DMSO (*) or PTEN KO mice treated with DMSO for 2 weeks (#). (**a**) Hematoxylin staining (H&E, bar 100 μm); H&E (M) indicates magnified photos (bar 100 μm). Oil red O staining (bar 40 μm), PI staining (bar 100 μm), and immunohistochemical staining of RIPK3 (bar 20 μm) and TUNEL (bar 100 μm) were performed. (**b**) The number of PI-positive cells. The frequency of PI-positive cells was compared to that in PTEN KO mice treated with DMSO for 2 weeks. (**c**) Serum GOT and GPT levels. The numbers of mice were as follows: Cnt (n = 10), KO-D (n = 13), KO-G (n = 10), KO-F (n = 11), KO-A (n = 14), and KO-R (n = 10). (**d**) The gene expression of proinflammatory cytokines and chemokines. Expression is shown as the fold increase relative to the control mice. Each group, n = 10. (**e**) The gene expression of profibrogenic factor. Expression is shown as the fold increase relative to te control mice. Each group, n = 10. (**f**) Body weight at 10 weeks of age (2-week treatment with each reagent). The numbers of mice were as follows: Cnt (n = 10), KO-D (n = 13), KO-G (n = 10), KO-F (n = 11), KO-A (n = 14), and KO-R (n = 10). No statistically significant (n.s) differences were observed. (**g**) The mean daily diet intake. No statistically significant (n.s) differences were observed.

We then examined the effects of apomorphine on an early stage of steatohepatitis. Apomorphine reduced the severity of steatosis and the number of PI-positive cells (Figure 2a,b). In addition, apomorphine reduced the serum transaminase and pro-inflammatory gene levels (Figure 2c,d). Although liver fibrosis was not evident in the histological assessment at 10 weeks of age, profibrogenic markers were decreased in number by apomorphine (Figure 2d). The magnitude of the favorable effects of apomorphine was similar to that of ferrostatin-1 (Figure 2c–e). In contrast, these favorable effects were not observed with GSK 872 (a necroptosis inhibitor) (Figure 2c–e). There were small changes in serum cholesterol and triglyceride levels regardless of treatment. 

We then ruled out the possibility of an anti-inflammatory effect of dopamine receptor agonists. Another dopamine receptor agonist, rotigotine, was also tested for its ability to reduce cell death. Although rotigotine reduced the severity of steatosis, it did not decrease the number of PI-positive cells, transaminase levels, and pro-inflammatory and pro-fibrogenic gene levels (Figure 2a–e). These data suggest that the effect of apomorphine does not depend on dopamine agonist activity.

To rule out the possibility of adverse effects of the reagents, we measured body weight and food intake. The body weight and dietary consumption of control and PTEN KO mice did not differ to a statistically significant extent, regardless of treatment (Figure 2f,g). 

### 3.4. Apomorphine Reduced the Oxidative Stress Marker but Not Iron Content in the Liver

**Figure 3:**
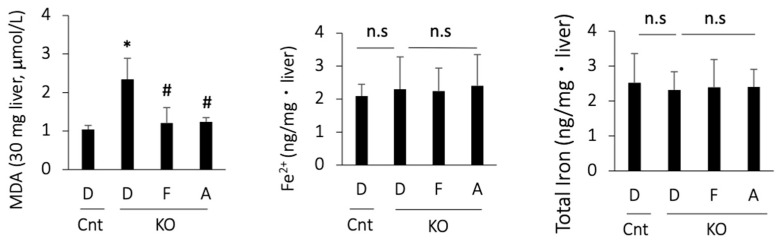
MDA and iron content in the liver. D, DMSO; F, ferrostatin-1; A, apomorphine. Cnt. control mice at 10 weeks of age; KO, PTEN KO mice at 10 weeks of age, n = 6 each group. # and * indicate statistically significant differences (*p* < 0.05) relative to the control mice treated with DMSO (*) or PTEN KO mice treated with DMSO for 2 weeks (#). n.s, not significant.

As there are no single biomarkers for ferroptosis, we assessed ferroptosis-associated events in PTEN KO mice. MDA, a product of lipid peroxidation, was increased in the PTEN KO mice and was suppressed by treatment with ferrostatin-1 or apomorphine (Figure 3). In contrast, hepatic iron content, including total iron and Fe^2+^, did not increase in the PTEN KO mice, irrespective of the treatment (Figure 3). 

### 3.5. Apomorphine Suppressed Cell Death through Ferroptosis

**Figure 4:**
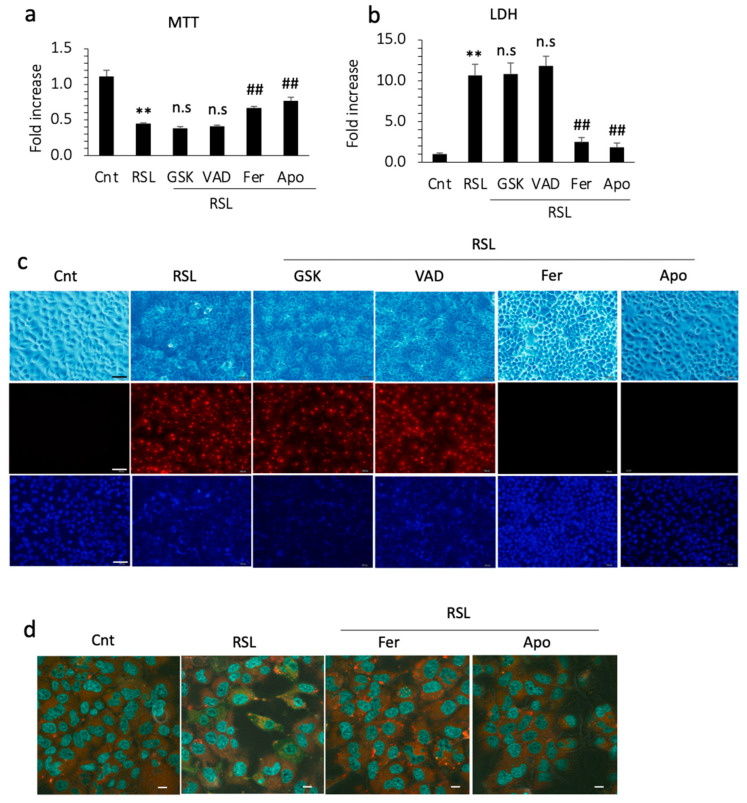
Huh 7 cells were cultured with each reagent for 1 h and then 0.5 μM RSL-3 was added. After 24 h, cells and supernatants were subjected to an MTT assay and LDH measurement, respectively. The concentrations of GSK, VAD, Ferrostain-1 (Fer), and apomorphine (Apo) were 5 μM, 1 μM, 1 μM, and 1 μM, respectively. (**a**) MTT assay. RSL treatment significantly reduced the number of living cells (**, *p* < 0.01). Fer and Apo treatment, but not GSK or VAD treatment, induced significant recovery from cell death (##, *p* < 0.01). (**b**) The LDH concentration in the supernatant. RSL treatment significantly increased the LDH level relative to the control (**, *p* < 0.01). Fer and Apo treatment, but not GSK or VAD treatment, significantly reduced LDH levels (##, *p* < 0.01). (**c**) Microscopic examination. Upper (light field), middle (PI staining), and lower (Hoechst33342). Bar 100 μm. (**d**) Lipid peroxidation using BODIPY581/591. Photographs were captured after incubation with ferrostatin-1 (Fer) or apomorphine (Apo) for 1 h and then with RSL-3 at 0.5 μM for 3 h. The cytoplasm of RSL-treated cells is green, indicating that lipid peroxidation occurred. Bar 50 μm. Data are the means of three independent experiments (**a**,**b**). n.s, not significant.

To confirm the inhibitory effect of apomorphine on ferroptosis, we performed cell death assays in vitro. RSL-3 (a ferroptosis inducer) successfully induced the cell death of Huh 7 cells within 24 h. In the cell viability assay and LDH levels in the supernatant, cell death was not inhibited by GSK872 and by an apoptosis inhibitor, Z-VAD-FMK. In contrast, cell death was inhibited by ferrostatin-1 and apomorphine (Figure 4a,b). Because Huh 7 cells are a hepatoma cell line, we also performed these experiments using primary cultured hepatocytes isolated from control and PTEN KO mice. The protective effects of ferrostatin-1 and apomorphine were observed in primary cultured hepatocytes (Supplemental Figure S1a,b). 

We also performed PI staining in the cell culture. PI staining was positive after RSL-3 treatment, which was not inhibited by necroptosis or apoptosis inhibitors (Figure 4c). In contrast, the cells were still negative for PI after pretreatment with ferrostatin-1 or apomorphine (Figure 4c). Thus, hepatocytes are PI-positive, even after ferroptosis. To further assess ferroptosis, we examined lipid peroxidation, a hallmark of ferroptosis. RSL-3 treatment induced lipid peroxidation in Huh 7 cells before cell death. This lipid peroxidation was inhibited by ferrostatin-1 and apomorphine (Figure 4d). 

### 3.6. Apomorphine Had Potent Radical Trapping Activity with an Inhibitory Effect of Cell Death

**Figure 5:**
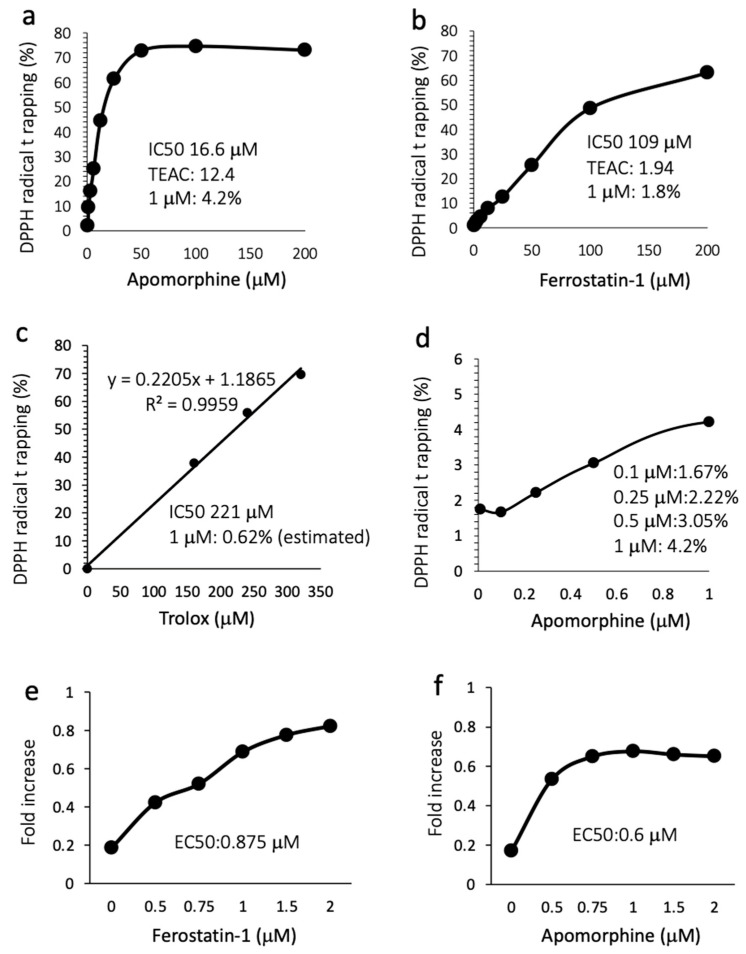
Radical trapping assay and the inhibitory effect against cell death. TEAC, Trolox-equivalent antioxidant capacity. (**a**) Apomorphine (0–200 μM). (**b**) Ferrostatin-1 (1–200 μM). (**c**) Trolox (0–350 μM). (**d**) Apomorphine (0.1–1.0 μM). (**e**,**f**) The cell death assay for ferrostain-1 (**e**) and apomorphine (**f**) was conducted using an MTT assay (RSL-3 at 1 μM). Data are representative of three independent experiments (duplicated in each assay) (**a**–**f**).

Because the mechanism by which apomorphine inhibits ferroptosis is largely unknown, we performed a radical rapping assay. The IC50s for DPPH radical trapping activity of apomorphine and ferrostatin-1 were 16.6 μM (4.4 μg/mL) and 109 μM (28.6 μg/mL), respectively (Figure 5a,b). The Trolox-equivalent antioxidant capacity in apomorphine and ferrostatin-1 was 12.4-fold and 1.94-fold higher, respectively. The DPPH removal capacities of apomorphine, ferrostatin-1, and Trolox at 1 μM were 4.2%, 1.8%, and 0.62% (estimated), respectively (Figure 5a–c). We additionally examined the DPPH trapping capacity of apomorphine at low concentrations. Apomorphine < 1 μM still exhibited radical trapping activity in comparison to Trolox at 1 μM (Figure 5d). These data indicate that the radical trapping activity of apomorphine was stronger than that of ferrostain-1 and Trolox (well-known radical trapping agents) at the same concentration. Indeed, when Huh 7 cells were cultured with RSL-3 at 1 μM, the EC50 values of ferrostatin and apomorphine were 0.875 and 0.6 μM, respectively. 

### 3.7. Apomorphine Activates nrf2

**Figure 6:**
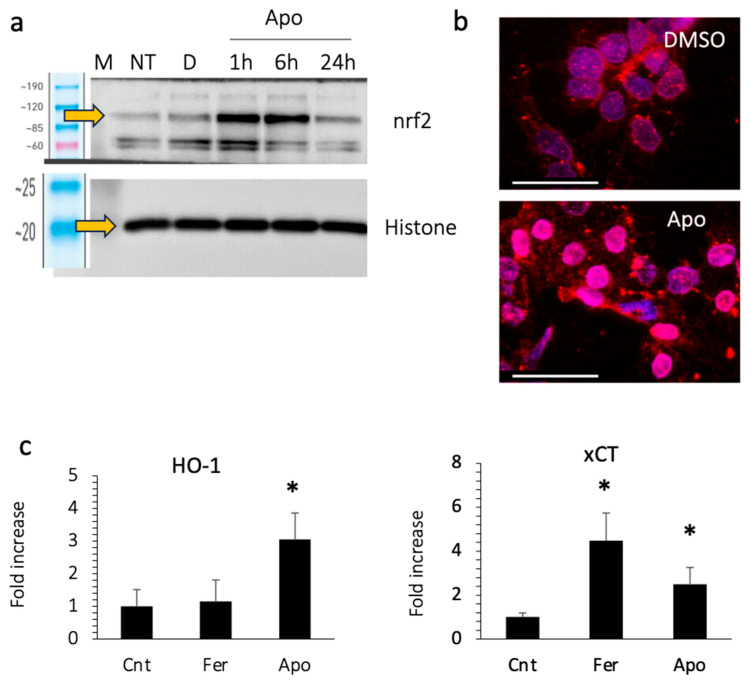
Huh 7 cells were treated with DMSO (control) or apomorphine (Apo) at 1 μM. (**a**) Western blotting. M, marker; NT, non-treatment. (**b**) Immunocytochemical staining of nrf2 (Bar 50 μm). The Huh 7 cells were treated with DMSO (control) or apomorphine (Apo) at 1 μM for 3 h. (**c**) The gene expression of downstream molecules of nrf2. Huh 7 cells were cultured with DMSO (Cnt), ferrostatin-1 (Fer) at 1 μM, or apomorphine (Apo) at 1 μM for 6 h. Moreover, 18S was used as an internal control. *, *p* < 0.05 relative to the control. Data are the means of three independent experiments (**c**).

We then examined the expression of nuclear nrf2, a transcription factor that responds to oxidative stress. Apomorphine treatment increased the expression of nrf2 in the nucleus, as well as the translocation of nrf2 to the nucleus at 1–6 h after stimulation (Figure 6a,b). We also examined the expression of the downstream genes of nrf2, including HO-1 and xCT (Figure 6c). The expression of these genes increased in cells treated with apomorphine. 

## 4. Discussion

We demonstrated ferroptosis in gene-modified mice and that it occurred at an early stage of steatohepatitis. In addition, apomorphine ameliorates steatohepatitis by inhibiting hepatocyte ferroptosis.

Ferroptosis has received considerable attention for its role in the pathogenesis of steatotic liver diseases. Earlier studies showed that ferroptosis was noted in steatohepatitis induced by methionine and/or a choline-deficient diet, in which mice characteristically showed body weight loss and increased insulin sensitivity [15,16,17]. Recent studies have shown that hepatocyte ferroptosis is also induced in mice fed a high-fat diet, characterized by obesity and insulin resistance [29,30]. In this study, we demonstrated that ferroptosis was induced in PTEN KO mice without modification of dietary components. These data indicated that ferroptosis is a mode of cell death in a wide range of steatohepatitis models.

We also demonstrated that ferroptosis occurred at an early stage of steatohepatitis in PTEN KO mice, in contrast with apoptosis and necrosis/necroptosis. The number of PI-positive cells increased at 10 weeks of age, indicating that cell death occurs at an early stage of steatohepatitis. Ferroptosis inhibitors, including apomorphine, decreased the number of PI-positive cells. In culture experiments, a ferroptosis inducer increased the number of PI-positive cells, which were suppressed by ferrostatin-1 and apomorphine. At an early stage of steatohepatitis (10 weeks of age), there were few apoptotic and necrotic/necroptotic hepatocytes. In contrast, approximately 5% and 4.5% of hepatocytes undergo apoptosis and necrosis/necroptosis, respectively, at an advanced stage of steatohepatitis (48 weeks of age). In addition, hepatocyte necrosis, characterized by ballooning, was noted in all fields of view at 48 weeks but not 10 weeks of age. These data indicate that ferroptosis is the initial event of cell death in our steatohepatitis model. This finding was also noted in another steatohepatitis model [17].

Apomorphine has a wide spectrum of dopamine receptor agonist activity, particularly dopamine receptor D2. Dopamine and a dopamine receptor agonist suppressed inflammation in a liver injury model induced by a combination of lipopolysaccharide and d-galactosamine [25,31]. To rule out the possibility that dopamine receptor agonists act in our steatohepatitis model, we tested rotigotine, a dopamine receptor D1–D5 agonist. However, rotigotine failed to reduce serum transaminase levels and proinflammatory gene expression. The activation of the dopamine D2 receptor has been reported to contribute to liver fibrosis by activating pro-fibrotic macrophages [32]. Apomorphine did not promote fibrosis in this model. In addition, apomorphine has been reported to cause adverse gastrointestinal events [18]. However, apomorphine did not reduce food intake, suggesting that adverse gastrointestinal events did not occur. Recently, we reported that apomorphine inhibited cell death without dopamine receptor agonist activity [23]. These data demonstrate that apomorphine can exert beneficial effects without dopamine receptor agonist activity. Thus, we considered that the beneficial effects of apomorphine were dependent on its inhibitory effect on ferroptosis.

Little information is available regarding the mechanism by which apomorphine inhibits ferroptosis. Previous reports have shown that apomorphine possesses radical trapping activity [20]. We revealed that apomorphine has potent radical trapping activity in comparison with ferrostatin-1 and Trolox, which are well-known radical trapping agents. The plasma concentration of apomorphine was 50–100 nM in the treatment of Parkinson's disease [20]. In our mouse experiments, the maximum concentration of apomorphine reached 3 μM after the intraperitoneal injection of 0.5 mg/kg body weight (manuscript under preparation). Thus, the concentration of apomorphine in our experiments was similar to that in actual use. In addition, apomorphine has been reported to activate nrf2 [21], a key molecule involved in oxidative stress. Apomorphine can increase reactive oxygen species (ROS) quantity in the cytoplasm, in addition to its function as a radical scavenger [33]. Apomorphine may release a small amount of ROS in the cytoplasm, which activates nrf2 but does not damage the cells. Although a high concentration of apomorphine (20 μM) is required for the activation of nrf2 in neuron cells [33], apomorphine can activate nrf2 at a low concentration (1 μM) in hepatocytes. Thus, apomorphine has the potential to show anti-ferroptosis effects at low doses in liver diseases, including MASLD.

Ferroptosis has two distinct functions. Ferroptosis inducers have been developed as cancer-killing agents. On the other hand, ferroptosis inhibitors have been developed to suppress inflammation by inhibiting cell death. One question arises: Can ferroptosis inhibitors be used for the treatment of steatohepatitis? In the present study, we used PTEN KO mice in which steatohepatitis developed, followed by liver fibrosis and cancer. In our preliminary experiments, 2-week treatment with a ferroptosis inhibitor did not show any specific effects in 48-week-old PTEN KO mice. This is probably due to the short duration of treatment. In addition, other types of cell death, including apoptosis and necrosis/necroptosis, may mask the cell death caused by ferroptosis. Recently, Cho et al. reported that hepatocyte ferroptosis activates hepatic stellate cells, a major source of extracellular matrix, and promotes liver fibrosis [34]. He et al. reported that the inhibition of ferroptosis reduced liver damage and inflammation, resulting in the suppression of liver cancer [35]. In addition, ferroptotic cells can transmit lipid peroxidation and subsequent cell death to neighboring cells [36]. Based on these reports and our data, we believe that ferroptosis inhibitors are useful at an early stage of steatohepatitis when there is less fibrosis and no cancer.

Although we demonstrated the beneficial effects of ferroptosis inhibition in a steatohepatitis model, the present study is associated with a couple of limitations. While we demonstrated that ferroptosis inhibition was effective for an early stage of steatohepatitis, we do not have sufficient data on its effect in advanced-stage steatohepatitis where liver fibrosis and liver cancer are established. Ferroptosis of hepatic stellate cells is associated with the resolution of liver fibrosis [37]. Thus, treatment with ferroptosis inhibitors in the advanced stage may promote liver fibrosis. In addition, the inhibition of ferroptosis may promote cancer progression by inhibiting cell death. In our experiments, apomorphine reduced the expression of profibrogenic genes at an early stage of steatohepatitis. In addition, 2-week treatment with ferroptosis inhibitors did not result in the growth of tumors in an advanced stage of steatohepatitis. Another limitation of the present study was the dopamine receptor agonist activity of apomorphine, which may have induced unexpected adverse events. Thus, we are now attempting to generate apomorphine derivatives that lose their dopamine receptor agonist activity. Further studies are necessary to determine the appropriate application of apomorphine in the treatment of steatotic liver disease.

In conclusion, ferroptosis occurs in steatohepatitis in PTEN KO mice, irrespective of diet. In addition, ferroptosis occurs at an early stage in steatohepatitis. Furthermore, apomorphine inhibited ferroptosis in hepatocytes at low concentrations. Therefore, it is worth testing the effects of apomorphine in human diseases related to ferroptosis, including MASLD.

## Data Availability

All data are included within the manuscript.

## Supplementary Materials

The following supporting information can be downloaded at: https://www.mdpi.com/article/10.3390/antiox13070805/s1. Figure S1: MTT assay and LDH measurement. MTT assay and LDH measurement in the supernatant of primary cultured hepatocytes isolated from WT mice (a,b) and PTEN KO mice (c,d) **, *p* < 0.01 vs. control; #, *p* < 0.05, ##, *p* < 0.01 vs. RSL group. Table S1: Sequence of primers for quantitative real-time PCR.
