# Supplementary material for: Apomorphine Suppresses the Progression of Steatohepatitis by Inhibiting Ferroptosis
Source: Antioxidants (Basel). 2024 Jul 2;13(7):805. doi: 10.3390/antiox13070805 (PMC11273851; doi:10.3390/antiox13070805)
Supplement: Supplementary file 1 [file antioxidants-13-00805-s001.zip › antioxidants-3087248-supplementary/RevTable S1.pdf]

Table S1. Sequence of primers for quantitative real-time PCR

Primers	Forward	Reverse	GenBank Accession number	NCBI Gene ID
18S	AGTCCCTGCCCTTTGTACACA	CGATCCGAGGGCCTCACTA	NR_003278	19791
Mouse Tnf	AGGGTCTGGGCCATAGAACT	CCACCACGCTCTTCTGTCTAC	NM_013693	21926
Mouse CCl2	ATTGGGATCATCTTGCTGGT	CCTGCTGTTCACAGTTGCC	NM_011333	20296
Mouse Collagen 1α1	TAGGACTGACCAAGGTGGCT	GGAACCTGGTTTCTTCTCACC	NM_007742	12842
Mouse TIMP-1	AGGTGGTCTCGTTGATTTCT	GTAAGGCCTGTAGCTGTGCC	NM_001044384	21857
Human HO-1	AAGACTGCGTTCCTGCTCAAC	AAAGCCCTACAGCAACTGTCG	NM_002133	3162
Human xCT	TCTCCAAAGGAGGTTACCTGC	AGACTCCCCTCAGTAAAGTGAC	NM_014331	23657
