# Supplementary figures and images for: Apomorphine Suppresses the Progression of Steatohepatitis by Inhibiting Ferroptosis
Source: Antioxidants (Basel). 2024 Jul 2;13(7):805. doi: 10.3390/antiox13070805 (PMC11273851; doi:10.3390/antiox13070805)

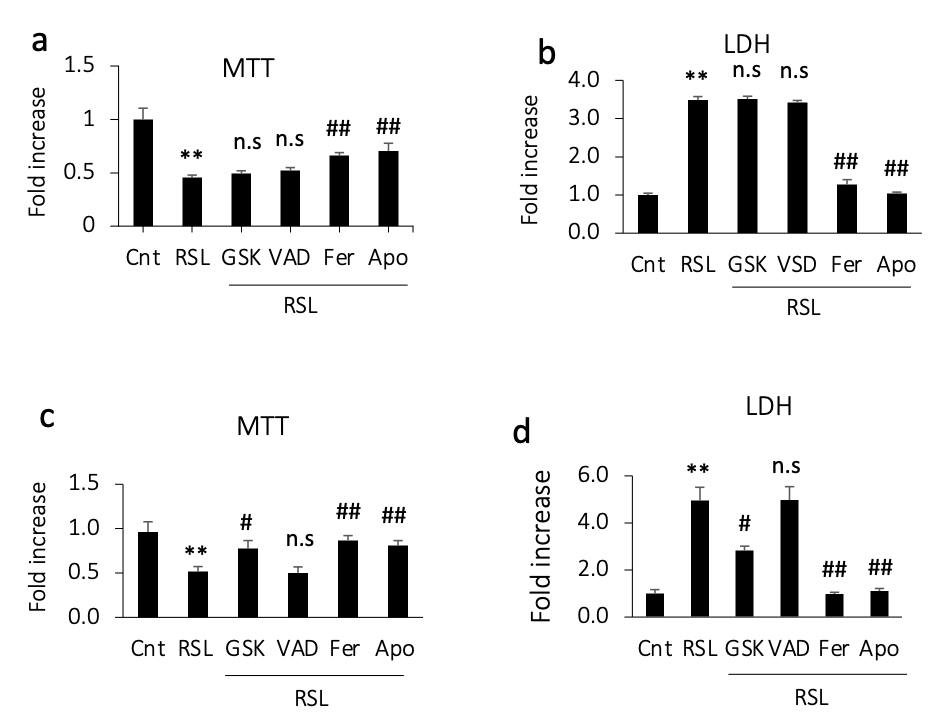

Supplement: Supplementary file 1 [file antioxidants-13-00805-s001.zip › antioxidants-3087248-supplementary/Miura Figure S1.tiff]
